# HLA class II variants defined by next generation sequencing are associated with sarcoidosis in Korean patients

**DOI:** 10.1038/s41598-022-13199-w

**Published:** 2022-06-03

**Authors:** Kateřina Sikorová, Su-Jin Moon, Hee-Young Yoon, Adam Strnad, Jin Woo Song, Martin Petrek

**Affiliations:** 1 Department of Pathological Physiology & Institute of Molecular and Translational Medicine, Faculty of Medicine and Dentistry, Palacky University Olomouc, Olomouc, Czech Republic; 2 Department of Pulmonary and Critical Care Medicine, Asan Medical Center, University of Ulsan College of Medicine, Seoul, Republic of Korea; 3 Division of Allergy and Respiratory Diseases, Soonchunhyang University Seoul Hospital, Seoul, Republic of Korea; 4 Department of Pathological Physiology, Faculty of Medicine and Dentistry, Palacky University Olomouc, Olomouc, Czech Republic

**Keywords:** Risk factors, Autoimmune diseases, Immunogenetics

## Abstract

Polymorphic genes with immune functions, namely those of the human leukocyte antigen (HLA) system, have been implicated in sarcoidosis pathogenesis. As HLA polymorphisms in sarcoidosis have not been yet investigated in the Korean population, we used next-generation sequencing (NGS), allowing detailed characterization of HLA alleles to investigate the role of HLA variation in Korean sarcoidosis patients. We enrolled 103 patients diagnosed by the ATS/ERS/WASOG guidelines at Asan Medical Centre, Seoul, Korea. Among those, genotyping of 7 HLA loci (HLA-A, -B, -C, -DQA1, -DQB1, -DRB1, -DPB1) was performed using Omixon Holotype™ kit and HLATwin software™. HLA allele frequencies were compared with frequency data on healthy Koreans from the allelic frequency databases, and 4-digit characteristics of HLA genotyping were used. Associations were assessed by two-tailed Fischer’s exact test with correction for multiple comparisons. Variants previously associated with sarcoidosis risk (HLA-C*03:04, HLA-DRB1*12:01, HLA-DRB1*14:54) and a known protective variant HLA-DPB1*04:01, were associated with sarcoidosis in Koreans. Further, we suggest new HLA variants associated with sarcoidosis risk (e.g., HLA-DQA1*05:08) and novel protective variants HLA-DQB1*03:02 and HLA-DQA1*01:02 in Koreans. This first study of HLA variation in Korean patients with sarcoidosis by precise genotyping methodology reports data that could serve future meta-analyses on HLA variation’s role in sarcoidosis.

## Introduction

Altered immune response to the yet unknown antigen(s), influenced by genetic and environmental factors, is the hallmark of sarcoidosis pathogenesis. The presentation of sarcoidosis is remarkably diverse as it is the course of the disease. The disease presents in mild asymptomatic to progressive forms, which occur in 15–20% of patients^1^.

The occurrence and clinical course of sarcoidosis depend on ethnicity; it is higher and more severe in African Americans than in patients of European descent^2,3^. In the Asian population, a generally lower incidence of sarcoidosis was reported^4^; however, new cases have increased in the last few years—for example, in South Korea, with an incidence rate of 0.85 per 100000^5^. Therefore, it is beneficial to distinguish between sarcoidosis phenotypes in their early stages for diagnostics, subsequent treatment, and general patient management. To accomplish this, it can be helpful to investigate the genetic profile of the patients in the context of their immunogenetic backgrounds, such as human leukocyte antigen (HLA) allele variants, HLA coding region genes, and other genes related to immune response and granuloma formation.

The key role of HLA, the most polymorphic human genetic system, in susceptibility to sarcoidosis and in modifying its clinical course has already been shown. However, most studies have been performed on populations of European or Afro-American descent^6–8^. However, in the East Asian region, only a limited number of studies on Japanese populations have been conducted^9,10^. To our knowledge, HLA polymorphisms have not yet been investigated in Korean sarcoidosis patients. Therefore, we focused this study on investigating polymorphic HLA alleles in sarcoidosis and its clinical phenotypes in Koreans.

## Results

### Comparison of the distribution of HLA variants between patients and healthy population

This study analyzed seven HLA loci at allele level in 103 patients with sarcoidosis from the Republic of Korea. HLA class I and class II allele frequencies in patients were compared with allele frequency data from healthy Korean population.

**Figure 1:**
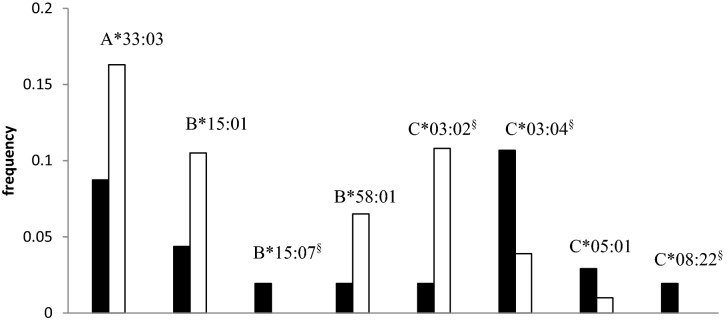
Frequencies of HLA class I alleles associated with sarcoidosis in Koreans. Patients—black columns, Control population—white columns. ^§^Significant *p*_*corr*_.

In the *HLA-A* locus, 21 different variants were determined by HLA genotyping (Table 1). One of these variants, HLA-A*33:03, occurred more often in the controls than in the patients, showing a protective association significant on the primary level. In the *HLA-B* locus, 45 different variants were distinguished (Table 1), and two of them, HLA-B*15:01 and B*58:01, were associated at the primary level as protective for sarcoidosis (Table 2). Further, HLA-B*15:07 was associated as a risk variant for sarcoidosis after correction for multiple comparisons (*p*_*corr*_ = 0.041). In the *HLA-C* locus, 22 different variants were distinguished (Table 1). HLA-C*05:01 was associated with sarcoidosis at a primary level as a risk variant. Protective variant HLA-C*03:02 (*p*_*corr*_ = 1.84 × 10^−04^) and risk variants HLA-C*03:04 (*p*_*corr*_ = 0.007) and HLA-C*08:22 (*p*_*corr*_ = 0.02) were associated after correction for multiple comparisons (Table 2, Fig. 1).

**Table 1:** Determined and associated alleles in seven HLA loci.

Locus	Determined variants	Associated on primary level	Associated after correction
HLA-A	21	1	0
HLA-B	45	3	1
HLA-C	22	4	3
HLA-DRB1	41	6	2
HLA-DQA1	17	8	3
HLA-DQB1	19	7	3
HLA-DPB1	18	6	4

Numbers of alleles that we were able to distinguish using NGS HLA genotyping and numbers of sarcoidosis associated alleles in seven HLA loci (primary level = before correction for multiple comparisons).

*HLA* human leukocyte antigen.

**Table 2:** HLA class I alleles associated with sarcoidosis in Korean patients (only the alleles associated at least on a primary level are shown).

HLA	OR [95% CI]	*p*-value	*p*_*corr*_
A*33:03	0.492 [0.294–0.821]	0.005	0.100
B*15:01	0.389 [0.193–0.783]	0.005	0.218
B*15:07	NA	0.001	**0.041**
B*58:01	0.285 [0.102–0.791]	0.008	0.290
C*03:02	0.163 [0.06–0.449]	8.38 × 10^−06^	**1.84 × 10**^**–04**^
C*03:04	2.949 [1.703–5.105]	3.16 × 10^−04^	**0.007**
C*05:01	2.97 [1.061–8.314]	0.046	0.643
C*08:22	NA	0.001	**0.02**

*NA* not analyzed (no variants were present in one of the analyzed groups), *OR* odds ratio, *CI* confidence interval, *HLA* human leukocyte antigen.

Significant *p*_*corr*_ (*p*-value after correction for multiple comparisons) in bold.

**Figure 2:**
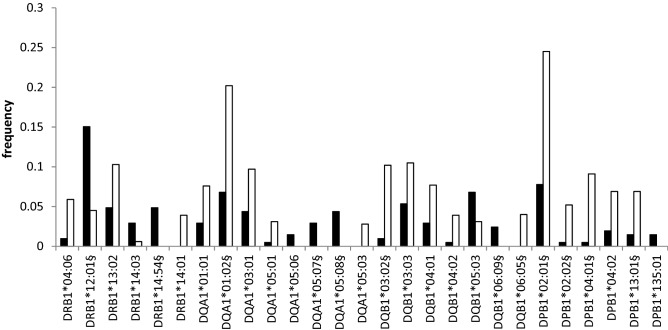
HLA Frequencies of HLA class II alleles associated with sarcoidosis in Koreans. Patients—black columns, Control population—white columns. ^§^Significant *p*_*corr*_.

*HLA-DRB1* is the HLA locus most often mentioned in connection with sarcoidosis; 41 different variants in this locus were determined by HLA genotyping in our patient group (Table 1). Variants HLA-DRB1*04:06, HLA-DRB1*13:02, HLA-DRB1*14:01 were protective and variant HLA-DRB1*14:03 acted as a risk allele for sarcoidosis, associations being significant on a primary level. After correction for multiple comparisons, alleles HLA-DRB1*14:54 (*p*_*corr*_ = 2.31 × 10^−05^) and HLA-DRB1*12:01 (*p*_*corr*_ = 9.12 × 10^−05^) were identified as risk factors for sarcoidosis (Table 3, Fig. 2).

**Table 3:** HLA class II alleles associated with sarcoidosis in Korean patients (only the alleles associated at least on a primary level are shown).

HLA	OR [95% CI]	*p*-value	*p*_*corr*_
DRB1*04:06	0.157 [0.037–0.654]	0.002	0.080
DRB1*12:01	3.754 [2.204–6.394]	2.22 × 10^–06^	**9.12** × **10**^**–05**^
DRB1*13:02	0.445 [0.224–0.881]	0.017	0.506
DRB1*14:03	4.955 [1.371–17.904]	0.016	0.483
DRB1*14:54	NA	5.63 × 10^–07^	**2.31** × **10**^**–05**^
DRB1*14:01	NA	0.001	0.056
DQA1*01:01	0.365 [0.154–0.865]	0.015	0.223
DQA1*01:02	0.288 [0.162–0.512]	2.14 × 10^–06^	**3.64** × **10**^**–05**^
DQA1*03:01	0.425 [0.207–0.87]	0.014	0.217
DQA1*05:01	0.152 [0.021–142]	0.037	0.474
DQA1*05:06	NA	0.014	0.211
DQA1*05:07	NA	1.86 × 10^–04^	**0.003**
DQA1*05:08	NA	2.42 × 10^–06^	**4.11** × **10**^**–05**^
DQA1*05:03	NA	0.01	0.161
DQB1*03:02	0.086 [0.021–0.356]	1.18 × 10^–06^	**2.25** × **10**^**–05**^
DQB1*03:03	0.481 [0.249–0.928]	0.027	0.404
DQB1*04:01	0.36 [0.152–0.851]	0.015	0.244
DQB1*04:02	0.12 [0.016–0.891]	0.01	0.169
DQB1*05:03	2.278 [1.13–4.592]	0.024	0.369
DQB1*06:09	NA	0.001	**0.015**
DQB1*06:05	NA	0.002	**0.029**
DPB1*02:01	0.259 [0.151–0.445]	3.28 × 10^–08^	**5.92** × **10**^**–07**^
DPB1*02:02	0.089 [0.012–0.654]	0.001	**0.017**
DPB1*04:01	0.049 [0.007–0.354]	1.08 × 10^–06^	**1.94** × **10**^**–05**^
DPB1*04:02	0.267 [0.095–0.753]	0.005	0.093
DPB1*13:01	0.199 [0.061–0.649]	0.002	**0.027**
DPB1*135:01	NA	0.014	0.222

*NA* not analyzed (no variants were present in one of the analyzed groups), *OR* odds ratio, *CI* confidence interval, *HLA* human leukocyte antigen.

Significant *p*_*corr*_ (*p*-value after correction for multiple comparisons) in bold.

Regarding the *HLA-DQA1* locus, 17 variants resulted from HLA genotyping (Table 1). HLA-DQA1*05:03, HLA-DQA1*03:01, HLA-DQA1*05:01, and HLA-DQA1*01:01 were associated on the primary level as protective for sarcoidosis. Variant HLA-DQA1*05:06 was associated with the risk for sarcoidosis on the primary level. After correction for multiple comparisons, HLA-DQA1*01:02 (*p*_*corr*_ = 3.64 × 10^−05^) was found as protective and HLA-DQA1*05:07 (*p*_*corr*_ = 0.003) and HLA-DQA1*05:08 (*p*_*corr*_ = 4.11 × 10^−05^) as risk factors (Table 3).

In the *HLA-DQB1* locus, 19 variants were determined (Table 1). Variants HLA-DQB1*03:03, HLA-DQB1*04:01, and HLA-DQB1*04:02 were associated as protective on the primary level, and variant HLA-DQB1*05:03, a risk factor for sarcoidosis. After correction for multiple comparisons, HLA-DQB1*03:02 (*p*_*corr*_ = 2.25 × 10^−05^) and HLA-DQB1*06:05 (*p*_*corr*_ = 0.029) were found as protective for sarcoidosis while risk variant was HLA-DQB1*06:09 (*p*_*corr*_ = 0.015); see Table 3.

In the *HLA-DPB1* locus, 18 different variants were characterized (Table 1). Variant HLA-DPB1*04:02 was considered protective on primary level and variant HLA-DPB1*135:01 associated with risk. After correction for multiple comparisons, variants HLA-DPB1*02:01 (*p*_*corr*_ = 5.92 × 10^−07^), HLA-DPB1*02:02 (*p*_*corr*_ = 0.017), HLA-DPB1*04:01 (*p*_*corr*_ = 1.94 × 10^−05^), and HLA-DPB1*13:01 (*p*_*corr*_ = 0.027) were found protective for sarcoidosis (Table 3).

Linkage disequilibrium between the alleles associated with sarcoidosis in Korean patients was also calculated. Non-randomly associated variants in HLA class II, HLA-DQA1*05:08–HLA-DRB1*12:01 (*p* = 3.33 × 10^−06^) and HLA-DQA1*05:07–HLA-DRB1*14:03 (*p* = 6.21 × 10^−10^) were found. The pair of linked variants in HLA class I, HLA-B*58:01–HLA-C*03:02 (*p* = 2.09 × 10^−07^), was observed (Table 4).

**Table 4:** Linkage disequilibrium between HLA alleles observed to be associated with sarcoidosis in Koreans.

HLA1	HLA2	*p*-value
B*58:01	C*03:02	2.09 × 10^–07^
DQA1*05:07	DRB1*14:03	6.21 × 10^–10^
DQA1*05:08	DRB1*12:01	3.33 × 10^–06^

*HLA* human leukocyte antigen.

### Comparison of the distribution of HLA variants in the context of different chest radiography stages

The association of HLA polymorphisms with CXR stage of the disease was analyzed. Patients were divided into two groups—the first group included 30 patients with CXR stage 0–1, and the second group included 73 patients with the more advanced CXR stages 2–4. HLA frequencies observed in the groups were compared with those of the control population. There were 12 variants associated with CXR stage 0–1 on primary level and two of them remained after correction for multiple comparisons (HLA-DRB1*12:01 *p*_*corr*_ = 0.033; HLA-DQA1*05:08 *p*_*corr*_ = 0.001) (Supplementary Table S1). The comparison between group CXR stages 2–4 and healthy controls resulted in 33 variants significantly associated with sarcoidosis at the primary level. After correction for multiple comparisons eight variants remained associated with more advanced forms of disease (HLA-B*15:07 *p*_*corr*_ = 0.013, HLA-C*03:04 *p*_*corr*_ = 0.007, HLA-C*08:22 *p*_*corr*_ = 0.006, HLA-DRB1*12:01 *p*_*corr*_ = 0.003, HLA-DRB1*14:54 *p*_*corr*_ = 4.57×10^−05^, HLA-DQA1*05:07 *p*_*corr*_ = 0.001, HLA-DQA1*05:08 *p*_*corr*_ = 0.003, HLA-DQB1*06:09 *p*_*corr*_ = 0.004) and seven were considered protective (HLA-A*33:03 *p*_*corr*_ = 0.038, HLA-C*03:02 *p*_*corr*_ = 0.005, HLA-DQA1*01:02 *p*_*corr*_ = 0.001, HLA-DQB1*03:02 *p*_*corr*_ = 3.86 × 10^−04^, HLA-DPB1*02:01 *p*_*corr*_ = 2.12 × 10^−05^, HLA-DPB1*02:02 *p*_*corr*_ = 0.021, HLA-DPB1*04:01 *p*_*corr*_ = 0.002) (Supplementary Table S2). Mutual comparison between CXR stage 0–1 patients (n = 30) and CXR stages 2–4 patients (n = 73) showed HLA-B*51:01 allele being associated with CXR 2-4 at the primary level (*p* = 0.0421); however, it did not attain significance after correction for multiple comparisons.

### HLA variants in patients with an extrapulmonary manifestation of sarcoidosis

Distribution of HLA polymorphisms in patients with extrapulmonary manifestation of sarcoidosis were compared with data from healthy controls. In this comparison, 30 variants were associated with extrapulmonary manifestation at the primary level (Supplementary Table S3). Eleven of them were significant also after correction for multiple comparisons, six as protective to extrapulmonary manifestation (HLA-A*33:03 *p*_*corr*_ = 0.038, HLA-C*03:02 *p*_*corr*_ = 0.019, HLA-DQA1*01:02 *p*_*corr*_ = 0.003, HLA-DQB1*03:02 *p*_*corr*_ = 3.86 × 10^−04^, HLA-DPB1*02:01 *p*_*corr*_ = 5.98 × 10^−06^, HLA-DPB1*04:01 *p*_*corr*_ = 0.002) and five as risk factors (HLA-B*15:07 *p*_*corr*_ = 0.013, HLA-C*08:22 *p*_*corr*_ = 0.047, HLA-DRB1*14:54 *p*_*corr*_ = 0.008, HLA-DQA1*05:08 *p*_*corr*_ = 0.003, HLA-DQB1*06:09 *p*_*corr*_ = 0.004). When mutual comparison of HLA frequencies in patients with and without extrapulmonary involvement was performed, four associations were revealed at the primary level (Supplementary Table S4); however, these did not attain significance after the correction.

### HLA variants in the context of clinical disease course (2 years follow up)

The patients were followed up at least 2 years after diagnosis. They were divided into three groups depending on the disease course as follows: patients whose disease had gone into remission, patients with stable disease, and patients whose disease progressed to a worse state. Next, the distribution of HLA in these three groups was compared with control population data. Two HLA variants are associated with a progressive, worsening course only at the primary level (Supplementary Table S5). Importantly, 39 variants were associated with disease improvement; ten of them remained significant after correction for multiple comparisons (Supplementary Table S6). Out of these ten stringently associated variants, five were associated with disease improvement (HLA-DRB1*12:01 *p*_*corr*_ = 0.006, HLA-DRB1*14:54 *p*_*corr*_ = 0.003, HLA-DQA1*05:07 *p*_*corr*_ = 1.86 × 10^−04^, HLA-DQA1*05:08 *p*_*corr*_ = 0.001, HLA-DQB1*06:09 *p*_*corr*_ = 0.010); remaining five variants were more frequent in population control group. In the group of patients with stable disease, 14 variants were associated on the primary level, and three of them attained significance after correction for multiple comparisons (Supplementary Table S7). Two of these three variants were associated with a stable disease course (HLA-DRB1*08:03 *p*_*corr*_ = 0.002 and HLA-DRB1*14:54 *p*_*corr*_ = 2.08 × 10^−04^); HLA-DPB1*02:01 was more frequent in the control group.

## Discussion

This study focused on immunogenetic profiling of Korean patients with sarcoidosis. It explored if variations in polymorphic HLA genes are related either to the disease itself or to several aspects of its clinical course. We report variants associated with the risk of sarcoidosis such as HLA-C*03:04, HLA-DRB1*12:01, HLA-DRB1*14:54, and a protective variant HLA-DPB1*04:01, previously described in other populations^6,7,11,12^, associated with sarcoidosis also in Koreans. Further, we suggest new HLA variants associated with sarcoidosis risk (e.g., HLA-DQA1*05:08) and novel protective variants HLA-DQB1*03:02 and HLA-DQA1*01:02 in Koreans. Finally, we confirm the previous reports that variants HLA-DRB1*12:01 and HLA-DPB1*04:01, linked to better prognosis^7,13^, are also valid for Koreans. In this context, we report a new association of HLA-DPB1*02:01 and -DPB1*02:02 with better disease prognosis in Korean patients.

In sarcoidosis, a multi-systemic granulomatous disease affecting mainly the lungs, alveolar CD4+ T cells present an unknown antigen interacting with HLA class II molecules. This role for HLA II can be one of the reasons for the observation that most of the HLA variants associated with sarcoidosis, also in our Korean patients, belong to HLA class II loci^3^.

*HLA-DRB1* locus has been widely associated with autoimmune diseases, including sarcoidosis^6,14^. We observed that HLA polymorphisms within this class II locus were associated with sarcoidosis also in Koreans. First, the variant HLA-DRB1*12:01 was detected as a risk factor more than threefold in the patients compared with the control population. In line with our finding, this variant was reported as susceptible in African American population, where it was associated with a doubled risk of disease^6,7^. We also observed that this variant was overrepresented in the patients who improved after 2 years of follow-up. Therefore, HLA-DRB1*12:01 may be associated with a better prognosis of sarcoidosis in Koreans. Second, we determined HLA-DRB1*14:54 as a risk factor for sarcoidosis in Koreans. Interestingly, from a limited number of studies on HLA in sarcoidosis in the East Asian population, Ishihara *et al*.^11^ reported that sarcoidosis is associated with HLA-DRB1*12:01 and also with alleles of the group HLA-DRB1*14 in Japanese patients. Further, alleles of the group HLA-DRB1*14, to which HLA-DRB1*14:54 belongs, have been previously associated with the risk of the disease, extensive extrapulmonary involvement, and the prolonged disease course in the Turkish population^15,16^.

*HLA-DQB1* was another HLA class II locus containing sarcoidosis-associated variant in Koreans, specifically HLA-DQB1*03:02 as a protective factor. This variant has not been mentioned in connection with sarcoidosis yet; nevertheless, it may have an important role in the immune response as a protective impact of this variant was mentioned in the context of HIV infection^17^.

*HLA-DQA1* locus has not been mentioned in connection with sarcoidosis very often. In our study, there was a linkage disequilibrium between *HLA-DQA1* alleles (e.g., HLA-DQA1*05) and associated *HLA-DRB1* variants, which could be the reason for the observed associations. Therefore, in the context of the disease course, we can nominate HLA-DQA1*01:02 as a protective factor against sarcoidosis in Koreans.

Regarding the *HLA-DPB1* locus, our observation of the over-representation of HLA-DPB1*04:01 in the Korean control population agrees with reports from Finnish sarcoidosis patients, where HLA-DPB1*04:01 was identified as a protective marker^12^. Further, when combined in the haplotype with HLA-DRB1*04:01, it was characteristic for spontaneous sarcoidosis resolution within 2 years^13^. In a wider context, the HLA-DPB1*04:01 variant was suggested as protective also in other autoimmune diseases, such as celiac disease^18^. In addition to HLA-DPB1*04:01, we characterized variants HLA-DPB1*02:01 and HLA-DPB1*02:02 as protective factors in Korean sarcoidosis patients.

Finally, regarding the *HLA-DPB1* locus, in our Korean cohort, HLA-DPB1*02:02 could be associated with a better sarcoidosis prognosis, as it did not occur in patients with CXR stage 2–4. In line with this speculation, HLA-DPB1*02:02 was associated with a better prognosis in another autoimmune condition—Graves’ disease in the Japanese population^19^.

HLA class I loci (*HLA-A*, *-B*, *-C*) have been previously rarely mentioned in connection with sarcoidosis and autoimmune diseases. In cases where HLA class I associations with sarcoidosis have been reported, either linkage disequilibrium between HLA class I and II or independent action in the context of immune response to intracellular mycobacterial infection were suggested for explanation^20,21^. As a protective factor and a risk factor, HLA-C*03:02 and HLA-C*03:04 were associated with sarcoidosis in Koreans, respectively. Moreover, HLA-C*03:04 was related to more advanced CXR stages and a worse prognosis. Another class I variant, HLA-A*33:03, was associated with protection from extrapulmonary involvement; this variant was previously observed with a frequency higher than 10% in the overall Korean population^22^.

Our study has limitations. Due to the smaller number of patients, some of the observed associations, especially in comparisons between clinical subgroups, could arise by chance and, therefore, should not be over-interpreted before independent replication. Ideally, the patient data should be compared with the data from a real, well-matched control population instead of the data from population databases. Although we could not use the preferred option due to pandemic limitations, the allelefrequencies.net database, from where we retrieved the control data for this study, has been considered an authoritative and reputable source representative of particular local/national populations^23^. Further, a high degree of ethnic homogeneity in the South Korean population^24^ is rather supporting than contradicting, using this kind of population control data for HLA allele frequency comparisons. However, we acknowledge that we could not use an ideally matched control population. Therefore, the observed associations should be interpreted cautiously until their verification in another cohort of Korean patients.

By contrast, there are important positive aspects of our study. So far, there have been only a few reports investigating the role of polymorphisms in HLA loci in sarcoidosis, where high resolution (i.e., very precise) genotyping methods were used^25^. However, the greatest strength of our study is that HLA in sarcoidosis has not been investigated in the Korean population at all, irrespective of precision level. Thus, our report provides the first data on the distribution of HLA variants in patients with sarcoidosis of this particular ethnicity, which is relevant to the importance of the ethnic concept of immunogenetics investigations in sarcoidosis^26^. Despite the above-discussed limitations, hereby reported high-resolution data can serve as a basis for future meta-analyses on the role of HLA variation in sarcoidosis.

## Conclusions

In this study, the first one about HLA polymorphisms and sarcoidosis in the Korean population, we extend associations between some HLA variants and sarcoidosis previously reported in other populations to Koreans. We suggest other new disease-associated variants, especially in the HLA class II loci, defined by NGS precise genotyping in our Korean patient cohort. However, before these associations’ plausible clinical significance or functional meaning can be investigated further, confirmatory (i.e., replication) studies in another Korean sarcoidosis patient cohort(s) are necessary. In the meantime, the reported data can be, however, useful for meta-analytical approaches to sarcoidosis genetics across ethnicities.

## Methods

### Patients

For this case–control study, 103 sarcoidosis cases (mean age ± SD, 46.9 ± 12.9 years; age range, 27–71 years; male [M]: female [F] ratio, 24: 79) were enrolled. All subjects were unrelated original inhabitants of South Korea diagnosed according to the ATS, ERS, and WASOG statement on sarcoidosis^27,28^ at Asan Medical Centre, Seoul, the Republic of Korea, from 17.5.1994 to 31.5.2019. 

Sarcoidosis cases were followed for 1.73–26.76 years after their diagnosis (median 11.95 years). After 2 years from the diagnosis, the patients were evaluated for the disease status as follows: improved (n = 58), stable disease (n = 32), and patients with progression of the disease (n = 5) for the remaining eight patients this information was missing due to loss to follow-up, death of the patients or other reasons. Concerning the treatment, 64 patients were treated with corticosteroids, in some cases in combination with methotrexate (n = 12), azathioprine (n = 4), or hydroxychloroquine sulphate (n = 4), and in one case in combination with mycophenolate mofetil. In two cases, the treatment with methotrexate was used independently without a combination of corticosteroids, and one case was treated with hydroxychloroquine sulphate only. Therefore, 36 patients did not receive any treatment.

Patients were also evaluated based on the Chest radiography stages (thoracic involvement) using Scadding’s criteria: stage 1 which involved bilateral hilar lymphadenopathy (BHL) only (n = 28), stage 2 for BHL with pulmonary infiltrates (n = 64), stage 3 with parenchymal infiltrates only (n = 6), stage 4 with pulmonary fibrosis (n = 3); two patients (stage 0) had normal radiograph.

Regarding extrapulmonary involvement, other organs than lungs have also been affected in some patients—eyes (n = 21), extra pulmonary lymph nodes (n = 19), skin (n = 16), spleen (n = 11), heart (n = 7), liver (n = 7), musculoskeletal manifestation (n = 2), renal affection (n = 2), neurosarcoidosis (n = 2), pancreas (n = 1), and hypercalcemia was presented in 34 patients.

### NGS HLA analysis

HLA genotypes for HLA-A, -B, -C, -DRB1, -DQA1, -DQB1, and -DPB1 loci were determined by high-resolution NGS technique using Omixon Holotype HLA kits configuration 96/7 v2 (Omixon Biocomputing Ltd, Budapest, Hungary) for library preparation and subsequent sequencing on Illumina MiSeq next-generation sequencing platform.

We worked with DNA isolated at Asan Medical Centre, Seoul, Korea. Blood samples were collected in EDTA-coated blood collection tubes (BD Vacutainer No. 367844). DNA in the blood was extracted using QIAamp DNA mini and Blood Kit (Qiagen No. 51306).

The HLA class I and class II loci were amplified by long-range PCR in separate samples in locus-specific 25 µl reactions, comprising 2.5 µl of PCR buffer, 1.25 µl of dNTP mix, 2 µl of locus-specific primers, 0.4 µl of LR PCR enzyme, and 5 µl of genomic DNA (35 ng/µl). A combined DQB1 enhancer (5.6 µl/sample) was added to the DQB1 master mix. The conditions for class I gene amplification on Mastercycler nexus thermal cycler (Eppendorf, Hamburg, Germany) were set as follows: 95 °C for 3 min, followed by 35 cycles of 95 °C for 15 s, 65 °C for 30 s, and 68 °C for 5 min, followed by final incubation at 68 °C for 10 min. For class II genes, the conditions were: 95 °C for 3 min, 35 cycles of 93 °C for 15 s, 60 °C for 30 s, and 68 °C for 9 min, followed by final incubation at 68 °C for 10 min. Amplicon presence was validated by 2% agarose gel electrophoresis. DNA was quantified using QuantiFluor fluorescent dsDNA staining system (Promega, Madison, Wisconsin, USA) and EnSpire Multimode plate reader (PerkinElmer, Waltham, MA, USA).

All seven amplicons from each sample were pooled into a final 35 µl volume on a new 96-well PCR plate and purified from residual primers and unincorporated nucleotides with the use of ExoSAP-iT enzyme mix (Affymetrix Inc., Santa Clara, CA, USA).

The next step was library preparation. The pooled amplicons were fragmented, followed by end repair and ligation with sample-specific indexed adaptors. Indexed sample-specific libraries were subsequently combined into a 900 µl pooled library volume and mixed with 900 µl of the AMPure XP beads (Beckman Coulter, Beverly, Massachusetts, USA) to carry out magnetic bead-based library clean-up. Pooled library fragments ranging between 650 and 1300 bp in size were subsequently selected on Agencourt AMPure XP beads. The concentration of the size selected library was determined on LightCycler 480 II (Roche Diagnostics, Mannheim, Germany) real-time PCR instrument using KAPA Sybr Fast qPCR Master Mix (KAPA Biosystems, Boston, Massachusetts, USA) and DNA standards ranging from 0.02 to 20 pM concentrations. Prior to sequencing, the library was diluted to a 2 nM concentration, denatured by NaOH, diluted with hybridization buffer, and the 9 pM library was loaded on MiSeq (Illumina, San Diego, CA, USA) and sequenced in a single 300-cycle (V2) paired-end sequencing run. Collected reads were exported in .fastq format.

Allele assignment for all seven loci was performed with the Omixon Twin software v4.2.0 and the IPD-IMGT/HLA database Release 3.39.0 (July 2020).

The laboratory where the HLA typing took place has a consistent record of passing international external proficiency testing (quality control) for HLA DNA genotyping since 2014. All experiments, from DNA extraction to the determination of HLA genotypes, were performed in accordance with relevant guidelines and regulations.

### Statistical analysis of the patient genotyping data

Comparison with a healthy population from Korea in HLA-A, -B, -C (HLA-class I) and HLA-DQA1,-DQB1, -DRB1, -DPB1 (HLA-class II) loci was performed. HLA allele frequencies obtained in patients were compared with frequencies of alleles determined in the corresponding loci in a healthy population from Korea. For HLA class I, population one from South Korea was selected as a healthy population, whereas for HLA class II, population three was used. Data for both populations were retrieved from the database allelefrequencies.net (http://allelefrequencies.net/pop6001c.asp?pop_id=1336; http://allelefrequencies.net/pop6001c.asp?pop_id=1870)^23^.

The association of HLA variants typed by NGS was validated by the two-tailed Fisher exact probability test to estimate the p-value, odds ratio (OR), and 95% confidence interval (CI). Correction for multiple comparisons was performed according to the following formula: 1 − (1 − *p*)^n^, where n = count of distinguished alleles in the loci. The statistical analysis was performed using http://vassarstats.net/odds2x2.html, https://www.graphpad.com/quickcalcs/contingency2/, and https://www.hiv.lanl.gov/content/immunology/hla/index.html.

The linkage disequilibrium calculation was also performed by software https://www.hiv.lanl.gov/content/immunology/hla/index.html. The number of two-way tests performed was 9636 (N2 = 9636). The *p*-value for two-way comparisons as 0.05/N2 = 5.189 × 10^−06^ for a 95% confidence level was calculated. As significant, results with p-values less than 5.189 × 10^−06^ were considered.

### Ethical approval

Patients provided informed consent with participation in the study, which was approved by the institutional review board (Institutional Review Board of Asan Medical Center IRB No—2018-2170).

## Supplementary Information

Supplementary Tables.

## Data Availability

The complete data, which served as the basis for analyses presented in the manuscript, including the data on the distribution of all detected HLA alleles in the investigated Korean patients with sarcoidosis (Supplementary Table S8), are provided within the Supplementary Information file.
