# Supplementary material for: HLA class II variants defined by next generation sequencing are associated with sarcoidosis in Korean patients
Source: Sci Rep. 2022 Jun 3;12:9302. doi: 10.1038/s41598-022-13199-w (PMC9166778; doi:10.1038/s41598-022-13199-w)
Supplement: Supplementary file 1 — Supplementary Tables. [file 41598_2022_13199_MOESM1_ESM.pdf]

SUPPLEMENTARY INFORMATION:

HLA class II variants defined by next generation sequencing are associated with sarcoidosis in Korean patients

Kateřina Sikorová[1], Su-Jin Moon[2], Hee-Young Yoon[3], Adam Strnad[4], Jin Woo Song[2]*, Martin Petrek[1]*

[1] Department of Pathological Physiology & Institute of Molecular and Translational Medicine; Faculty of Medicine and Dentistry, Palacky University Olomouc, Olomouc, Czech Republic
[2]Department of Pulmonary and Critical Care Medicine, Asan Medical Center, University of Ulsan College of Medicine, Seoul, Republic of Korea
[3]Division of Allergy and Respiratory Diseases, Soonchunhyang University Seoul Hospital, Seoul, Republic of Korea
[4] Department of Pathological Physiology, Faculty of Medicine and Dentistry, Palacky University Olomouc, Olomouc, Czech Republic
***Correspondence: MP: martin.petrek2@fnol.cz; Tel.: +420 585632501; JWS: jwsongasan@gmail.com

**Allele**	***f* case**	***f* ctrl**	**OR [95% CI]**	***p*-value**	p_{corr}
HLA-A*02:07	0.08	0.03	2.94(1.1-7.89)	0.043	0.601
HLA-C*03:02	0.02	0.11	0.14(0.02-1.02)	0.016	0.294
HLA-DRB1*12:01§	0.17	0.05	4.24(1.95-9.19)	0.001	**0.033**
HLA-DRB1*14:54	0.03	0	NA	0.007	0.252
HLA-DQA1*01:02	0.07	0.2	0.28(0.1-0.79)	0.009	0.145
HLA-DQA1*03:01	0.02	0.1	0.16(0.02-1.16)	0.033	0.436
HLA-DQA1*05:08§	0.07	0	NA	$4.7*10^{-05}$	**0.001**
HLA-DQB1*03:02	0.02	0.1	0.15(0.02-1.09)	0.034	0.485
HLA-DPB1*02:01	0.08	0.25	0.28(0.11-0.71)	0.004	0.062
HLA-DPB1*04:01	0	0.09	NA	0.006	0.110
HLA-DPB1*04:02	0	0.07	NA	0.026	0.375
HLA-DPB1*135:01	0.03	0	NA	0.007	0.120

Supplementary Table S1. HLA alleles associated with CXR stages 0-1(n=30) in Korean patients with sarcoidosis compared with population control data (only the alleles associated at least on primary level are shown).

f case-allele frequency in the patient group
f ctrl-allele frequency in the control group
OR- odds ratio, CI-confidence interval
§- significant p_{corr} (*p*- value after correction for multiple comparisons) in bold
NA- not analysed (in one of the analysed groups there were no variants present)

**Allele**	***f* case**	***f* ctrl**	**OR [95% CI]**	***p*-value**	p_{corr}
HLA-A*33:03§	0.07	0.16	0.38(0.19-0.73)	0.002	**0.038**
HLA-B*15:01	0.05	0.11	0.43(0.2-0.94)	0.035	0.798
HLA-B*15:07§	0.03	0	NA	$2.83*10^{-04}$	**0.013**
HLA-B*51:01	0.14	0.08	1.73(1.03-2.92)	0.046	0.877

HLA-B*58:01	0.02	0.07	0.3(0.09-0.97)	0.036	0.812
HLA-C*03:02§	0.02	0.11	0.17(0.05-0.55)	$2.41*10^{-04}$	**0.005**
HLA-C*03:04§	0.12	0.04	3.25(1.78-5.93)	$3.13*10^{-04}$	**0.007**
HLA-C*05:01	0.03	0.01	3.51(1.18-10.48)	0.036	0.553
HLA-C*08:22§	0.03	0	NA	$2.83*10^{-04}$	**0.006**
HLA-DRB1*01:01	0.02	0.06	0.31(0.1-1.02)	0.044	0.842
HLA-DRB1*03:01	0	0.03	NA	0.033	0.750
HLA-DRB1*04:06	0.01	0.06	0.22(0.05-0.93)	0.021	0.576
HLA-DRB1*12:01§	0.14	0.05	3.56(1.97-6.44)	$7.83*10^{-05}$	**0.003**
HLA-DRB1*12:10	0.01	0	NA	0.034	0.754
HLA-DRB1*13:02	0.04	0.1	0.37(0.16-0.88)	0.017	0.503
HLA-DRB1*14:03	0.04	0.01	7.08(1.95-25.66)	0.004	0.147
HLA-DRB1*14:54§	0.05	0	NA	$1.11*10^{-06}$	**$4.57*10^{-05}$**
HLA-DRB1*14:01	0	0.04	NA	0.008	0.283
HLA-DQA1*01:01	0.03	0.08	0.34(0.12-0.97)	0.042	0.514
HLA-DQA1*01:02§	0.07	0.2	0.29(0.15-0.57)	$6.13*10^{-05}$	**0.001**
HLA-DQA1*05:01	0	0.03	NA	0.035	0.454
HLA-DQA1*05:06	0.01	0	NA	0.034	0.441
HLA-DQA1*05:07§	0.04	0	NA	$3.55*10^{-05}$	**0.001**
HLA-DQA1*05:08§	0.03	0	NA	$1.99*10^{-04}$	**0.003**
HLA-DQB1*02:01	0	0.03	NA	0.033	0.474
HLA-DQB1*03:02§	0.01	0.1	0.06(0.01-0.44)	$2.03*10^{-05}$	**$3.86*10^{-04}$**
HLA-DQB1*06:09§	0.03	0	NA	$1.98*10^{-04}$	**0.004**
HLA-DQB1*06:05	0	0.04	NA	0.008	0.143
HLA-DPB1*02:01§	0.08	0.25	0.25(0.13-0.48)	$1.18*10^{-06}$	**$2.12*10^{-05}$**
HLA-DPB1*02:02§	0	0.05	NA	0.001	**0.021**
HLA-DPB1*04:01§	0.01	0.09	0.07(0.01-0.5)	$8.40*10^{-05}$	**0.002**
HLA-DPB1*13:01	0.01	0.07	0.19(0.04-0.78)	0.006	0.105
HLA-DPB1*15:01	0.01	0	NA	0.034	0.460

Supplementary Table S2. HLA alleles associated with CXR stages 2–4 (n=73) in Korean patients with sarcoidosis compared with population control data (only the alleles associated at least on primary level are shown).
For the legend see Supplementary Table S1

Allele	*f* case	*f* ctrl	OR [95% CI]	*p*-value	p_{corr}
HLA-A*33:03§	0.07	0.16	0.38(0.19-0.73)	0.002	**0.038**
HLA-B*15:07§	0.03	0	NA	$2.82*10^{-04}$	**0.013**
HLA-B*44:02	0.04	0.01	3.54(1.3-9.64)	0.022	0.631
HLA-B*55:02	0	0.03	NA	0.039	0.832
HLA-C*03:02§	0.03	0.11	0.23(0.08-0.64)	0.001	**0.019**
HLA-C*03:04	0.1	0.04	2.82(1.51-5.28)	0.002	0.054
HLA-C*05:01	0.04	0.01	4.24(1.51-11.92)	0.012	0.230
HLA-C*08:22§	0.02	0	NA	0.002	**0.047**
HLA-DRB1*04:06	0.01	0.06	0.11(0.01-0.81)	0.005	0.187
HLA-DRB1*12:01	0.12	0.05	2.79(1.49-5.23)	0.002	0.097
HLA-DRB1*12:10	0.01	0	NA	0.034	0.754
HLA-DRB1*14:54§	0.03	0	NA	$1.99*10^{-04}$	**0.008**
HLA-DRB1*14:01	0	0.04	NA	0.008	0.283
HLA-DQA1*01:02§	0.08	0.2	0.32(0.17-0.61)	$1.78*10^{-04}$	**0.003**
HLA-DQA1*05:07	0.02	0	NA	0.006	0.099

HLA-DQA1*05:08§	0.03	0	NA	$1.99*10^{-04}$	**0.003**
HLA-DQB1*03:02§	0.01	0.1	0.06(0.01-0.44)	$2.03*10^{-05}$	**$3.86*10^{-04}$**
HLA-DQB1*04:01	0.03	0.08	0.34(0.12-0.95)	0.029	0.426
HLA-DQB1*04:02	0	0.04	NA	0.008	0.143
HLA-DQB1*05:03	0.09	0.03	3.05(1.48-6.29)	0.004	0.078
HLA-DQB1*06:03	0.04	0.01	3.87(1.28-11.65)	0.019	0.312
HLA-DQB1*06:09§	0.03	0	NA	$1.99*10^{-04}$	**0.004**
HLA-DQB1*06:05	0	0.04	NA	0.008	0.143
HLA-DPB1*02:01§	0.07	0.25	0.23(0.12-0.44)	$3.32*10^{-07}$	**$5.98*10^{-06}$**
HLA-DPB1*02:02	0.01	0.05	0.13(0.02-0.93)	0.012	0.199
HLA-DPB1*04:01§	0.01	0.09	0.07(0.01-0.5)	$8.40*10^{-05}$	**0.002**
HLA-DPB1*04:02	0.01	0.07	0.19(0.04-0.78)	0.006	0.105
HLA-DPB1*13:01	0.01	0.07	0.19(0.04-0.78)	0.006	0.105
HLA-DPB1*135:01	0.02	0	NA	0.006	0.105
HLA-DPB1*15:01	0.01	0	NA	0.034	0.460

Supplementary Table S3. HLA alleles associated with extrapulmonary manifestation of sarcoidosis in Koreans (n=73) compared with population control data (only the alleles associated at least on primary level are shown).
For the legend see Supplementary Table S1

**Allele**	***f* (pulmonary only)**	***f* (extrapulmonary)**	***p*-value**	**p_{corr}**
HLA-A*02:06	0.1429	0.0455	0.0309	0.483
HLA-C*15:02	0.08	0.0072	0.0183	0.334
HLA-DRB1*12:01	0.2593	0.125	0.0301	0.714
HLA-DQA1*05:06	0.0682	0	0.0205	0.297

Supplementary Table S4. HLA alleles associated with extrapulmonary manifestation of sarcoidosis in Korean patients (n=73) compared with Korean patients with pulmonary involvement only (n=30); only the alleles associated on primary level are shown.
For the legend see Supplementary Table S1

**Allele**	***f* case**	***f* ctrl**	**OR [95% CI]**	***p*-value**	**p_{corr}**
HLA-DQB1*05:03	0.2	0.03	7.81(1.56-39.15)	0.041	0.548
HLA-DPB1*05:01	0	0.37	NA	0.016	0.257

Supplementary Table S5. HLA alleles associated with disease progression in Korean patients with sarcoidosis (n=5) compared with population control data; only the alleles associated on primary level are shown.
For the legend see Supplementary Table S1

**variant**	***f* case**	***f* ctrl**	**OR [95% CI]**	***p*-value**	**p_{corr}**
HLA-A*33:03§	0.06	0.16	0.33(0.15-0.72)	0.002	**0.049**
HLA-B*15:07	0.02	0	NA	0.011	0.401
HLA-B*44:02	0.05	0.01	4.51(1.65-12.31)	0.008	0.312
HLA-B*44:03	0.03	0.09	0.29(0.09-0.92)	0.026	0.699
HLA-C*03:02	0.03	0.11	0.22(0.07-0.7)	0.003	0.059
HLA-C*03:04	0.1	0.04	2.85(1.44-5.62)	0.004	0.094
HLA-C*05:01	0.05	0.01	5.4(1.91-15.23)	0.004	0.090
HLA-C*08:22	0.02	0	NA	0.011	0.222
HLA-DRB1*01:01	0.02	0.06	0.26(0.06-1.09)	0.048	0.869
HLA-DRB1*04:06	0	0.06	NA	0.002	0.088
HLA-DRB1*12:01§	0.15	0.05	3.64(1.93-6.86)	$1.49*10^{-04}$	**0.006**
HLA-DRB1*12:10	0.02	0	NA	0.023	0.613

HLA-DRB1*13:01	0.04	0.01	4.07(1.27-13)	0.024	0.634
HLA-DRB1*13:02	0.03	0.1	0.31(0.11-0.87)	0.015	0.459
HLA-DRB1*14:03	0.05	0.01	9.01(2.48-32.75)	0.001	0.054
HLA-DRB1*14:54§	0.04	0	NA	$7.49*10^{-05}$	**0.003**
HLA-DRB1*14:01	0	0.04	NA	0.023	0.610
HLA-DQA1*01:01	0.03	0.08	0.32(0.1-1.05)	0.047	0.557
HLA-DQA1*01:02§	0.06	0.2	0.25(0.12-0.56)	$1.13*10^{-04}$	**0.002**
HLA-DQA1*02:01	0.01	0.06	0.15(0.02-1.08)	0.031	0.418
HLA-DQA1*03:01	0.03	0.1	0.25(0.08-0.8)	0.011	0.168
HLA-DQA1*05:06	0.03	0	NA	0.003	0.057
HLA-DQA1*05:07§	0.05	0	NA	$1.10*10^{-05}$	**$1.86*10^{-04}$**
HLA-DQA1*05:08§	0.04	0	NA	$7.49*10^{-05}$	**0.001**
HLA-DQB1*02:02	0.01	0.05	0.15(0.02-1.12)	0.031	0.447
HLA-DQB1*03:02§	0.01	0.1	0.08(0.01-0.56)	$2.46*10^{-04}$	**0.005**
HLA-DQB1*04:01	0.03	0.08	0.32(0.1-1.04)	0.047	0.596
HLA-DQB1*04:02	0	0.04	NA	0.023	0.354
HLA-DQB1*06:03	0.04	0.01	4.07(1.27-13)	0.024	0.373
HLA-DQB1*06:04	0.01	0.06	0.13(0.02-0.95)	0.013	0.225
HLA-DQB1*06:09§	0.03	0	NA	0.001	**0.010**
HLA-DQB1*06:05	0	0.04	NA	0.023	0.356
HLA-DPB1*02:01§	0.09	0.25	0.32(0.17-0.62)	$1.55*10^{-04}$	**0.003**
HLA-DPB1*02:02	0.01	0.05	0.16(0.02-1.17)	0.031	0.432
HLA-DPB1*03:01	0	0.04	NA	0.037	0.497
HLA-DPB1*04:01§	0.01	0.09	0.09(0.01-0.63)	0.001	**0.011**
HLA-DPB1*04:02	0.02	0.07	0.24(0.06-0.99)	0.033	0.458
HLA-DPB1*13:01	0.02	0.07	0.24(0.06-0.99)	0.033	0.458
HLA-DPB1*15:01	0.02	0	NA	0.023	0.341

Supplementary Table S6. HLA alleles associated with disease improvement in Korean patients with sarcoidosis (n=58) compared with population control data (only the alleles associated at least on primary level are shown).
For the legend see Supplementary Table S1

variant	*f* case	*f* ctrl	OR [95% CI]	p-value	p_{corr}
HLA-B*15:07	0.03	0	NA	0.004	0.156
HLA-B*40:02	0.09	0.04	2.62(1.06-6.45)	0.044	0.869
HLA-C*03:02	0.02	0.11	0.13(0.02-0.95)	0.017	0.308
HLA-C*03:04	0.09	0.04	2.55(1.04-6.28)	0.049	0.667
HLA-C*08:22	0.03	0	NA	0.004	0.080
HLA-DRB1*08:03§	0.25	0.07	4.23(2.24-8.02)	$4.07*10^{-05}$	**0.002**
HLA-DRB1*12:01	0.13	0.05	3.03(1.32-6.93)	0.013	0.415
HLA-DRB1*14:54§	0.08	0	NA	$5.07*10^{-06}$	**$2.08*10^{-04}$**
HLA-DQA1*01:02	0.09	0.2	0.41(0.17-0.97)	0.044	0.538
HLA-DQA1*05:08	0.03	0	NA	0.008	0.127
HLA-DQB1*03:01	0.03	0.13	0.22(0.05-0.92)	0.024	0.368
HLA-DQB1*03:02	0.02	0.1	0.14(0.02-1.02)	0.022	0.348
HLA-DPB1*02:01§	0.06	0.25	0.21(0.07-0.57)	$4.67*10^{-04}$	**0.008**
HLA-DPB1*04:01	0	0.09	NA	0.007	0.115

Supplementary Table S7. HLA alleles associated with stable disease in Korean patients with sarcoidosis (n=32) compared with population control data (only the alleles associated at least on primary level are shown).
For the legend see Supplementary Table S1

Allele	Allele count	Allele frequency
A*01	2	0.01
A*01:01	4	0.019
A*02	6	0.03
A*02:01	38	0.184
A*02:03	3	0.015
A*02:06	14	0.068
A*02:07	10	0.049
A*02:10	1	0.005
A*02:110	1	0.005
A*03:01	5	0.024
A*03:02	1	0.005
A*11:01	20	0.097
A*24	4	0.019
A*24:02	47	0.228
A*26:01	7	0.034
A*26:03	4	0.019
A*30:01	4	0.019
A*30:04	3	0.015
A*31	6	0.029
A*31:01	8	0.039
A*33:03	18	0.087
B*07:02	5	0.024
B*08:01	1	0.005
B*13	2	0.01
B*13:01	3	0.015
B*13:02	3	0.015
B*14:01	2	0.01
B*15	2	0.01
B*15:01	9	0.044
B*15:02	1	0.005
B*15:07	4	0.019
B*15:11	7	0.034
B*15:18	2	0.01
B*27:05	3	0.015
B*35	2	0.01
B*35:01	12	0.058
B*35:02	1	0.005
B*35:03	1	0.005
B*37:01	2	0.01
B*38:02	4	0.019
B*39:01	2	0.01
B*40	2	0.01
B*40:01	9	0.044
B*40:02	12	0.058
B*40:03	1	0.005
B*40:06	5	0.024
B*44	2	0.01

B*44:02	6	0.029
B*44:03	12	0.058
B*46	2	0.01
B*46:01	13	0.063
B*48	2	0.01
B*48:01	11	0.053
B*51	4	0.019
B*51:01	22	0.107
B*51:02	1	0.005
B*52:01	8	0.039
B*54	2	0.01
B*54:01	11	0.053
B*55:02	1	0.005
B*55:04	1	0.005
B*56:01	1	0.005
B*57:01	1	0.005
B*58:01	4	0.019
B*59:01	3	0.015
B*67:01	2	0.01
C*01	18	0.087
C*01:02	34	0.165
C*01:03	1	0.005
C*02:02	1	0.005
C*03:02	4	0.019
C*03:03	29	0.141
C*03:04	22	0.107
C*04:01	9	0.044
C*05:01	6	0.029
C*06:02	6	0.029
C*07:02	12	0.058
C*07:04	1	0.005
C*07:06	4	0.019
C*08:01	11	0.053
C*08:02	2	0.01
C*08:03	2	0.01
C*08:22	4	0.019
C*12:02	7	0.034
C*12:03	1	0.005
C*14:02	19	0.092
C*14:03	8	0.039
C*15:02	5	0.024
DRB1*01	2	0.01
DRB1*01:01	6	0.029
DRB1*03	1	0.005
DRB1*03:01	1	0.005
DRB1*04:01	1	0.005
DRB1*04:03	4	0.019
DRB1*04:04	2	0.01

DRB1*04:05	13	0.063
DRB1*04:06	2	0.01
DRB1*04:07	1	0.005
DRB1*04:08	1	0.005
DRB1*04:10	1	0.005
DRB1*04:27	1	0.005
DRB1*07:01	9	0.044
DRB1*08	1	0.005
DRB1*08:02	1	0.005
DRB1*08:03	18	0.087
DRB1*09	4	0.019
DRB1*09:01	14	0.068
DRB1*11:01	8	0.039
DRB1*11:04	1	0.005
DRB1*12	4	0.019
DRB1*12:01	31	0.15
DRB1*12:02	4	0.019
DRB1*12:10	2	0.01
DRB1*12:58	1	0.005
DRB1*13:01	6	0.029
DRB1*13:02	10	0.049
DRB1*13:39	1	0.005
DRB1*14	2	0.01
DRB1*14:02	1	0.005
DRB1*14:03	6	0.029
DRB1*14:05	10	0.049
DRB1*14:07	2	0.01
DRB1*14:216	1	0.005
DRB1*14:54	10	0.049
DRB1*15	2	0.01
DRB1*15:01	10	0.049
DRB1*15:02	8	0.039
DRB1*15:140	2	0.01
DRB1*16:02	1	0.005
DQA1*01	24	0.117
DQA1*01:01	6	0.029
DQA1*01:02	14	0.068
DQA1*01:03	26	0.126
DQA1*01:04	17	0.083
DQA1*02:01	6	0.029
DQA1*03	24	0.117
DQA1*03:01	9	0.044
DQA1*03:02	22	0.107
DQA1*03:03	20	0.097
DQA1*04:01	1	0.005
DQA1*05:01	1	0.005
DQA1*05:05	14	0.068
DQA1*05:06	3	0.015

DQA1*05:07	6	0.029
DQA1*05:08	9	0.044
DQA1*06:01	4	0.019
DQB1*02:01	1	0.005
DQB1*02:02	6	0.029
DQB1*03	32	0.155
DQB1*03:01	22	0.107
DQB1*03:02	2	0.01
DQB1*03:03	11	0.053
DQB1*03:05	1	0.005
DQB1*04:01	6	0.029
DQB1*04:02	1	0.005
DQB1*05	18	0.087
DQB1*05:01	12	0.058
DQB1*05:02	10	0.049
DQB1*05:03	14	0.068
DQB1*06	19	0.092
DQB1*06:01	24	0.117
DQB1*06:02	8	0.039
DQB1*06:03	7	0.034
DQB1*06:04	7	0.034
DQB1*06:09	5	0.024
DPB1*02	33	0.16
DPB1*02:01	16	0.078
DPB1*02:02	1	0.005
DPB1*03:01	3	0.015
DPB1*04	9	0.044
DPB1*04:01	1	0.005
DPB1*04:02	4	0.019
DPB1*05	44	0.214
DPB1*05:01	70	0.34
DPB1*09	5	0.024
DPB1*09:01	2	0.01
DPB1*107:01	1	0.005
DPB1*13	7	0.034
DPB1*13:01	3	0.015
DPB1*135:01	3	0.015
DPB1*15:01	2	0.01
DPB1*48:01	1	0.005
DPB1*74	1	0.005

Supplementary Table S8.
Data on the occurrence of the alleles at the HLA-A, -B, -C, -DRB1, -DQA1, -DQB1 and -DPB1 loci, which we were able to distinguish by NGS HLA genotyping in samples from Korean patients with sarcoidosis (n=103).

Allele count – absolute number (A) of a given allele detected in the patient group, Allele frequency (*f*) – relative occurrence of a given allele (*f* = A / 2 x n)
